# Loss of myeloid differentiation protein 1 promotes atrial fibrillation in heart failure with preserved ejection fraction

**DOI:** 10.1002/ehf2.12620

**Published:** 2020-01-29

**Authors:** Wei Shuai, Bin Kong, Hongjie Yang, Hui Fu, He Huang

**Affiliations:** ^1^ Department of Cardiology Renmin Hospital of Wuhan University 238 Jiefang Road Wuhan Hubei 430060 China; ^2^ Cardiovascular Research Institute of Wuhan University Wuhan China; ^3^ Hubei Key Laboratory of Cardiology Wuhan China

**Keywords:** Myeloid differentiation protein 1, Fibrosis, Inflammation, Calcium handling proteins, Atrial fibrillation, Heart failure with preserved ejection fraction

## Abstract

**Aims:**

Myeloid differentiation protein 1 (MD1) is expressed in the mammalian heart and exerts an anti‐arrhythmic effect. Atrial fibrillation (AF) is closely related to heart failure with preserved ejection fraction (HFpEF). The potential impact of MD1 on AF vulnerability in an HFpEF model is not clear.

**Methods and results:**

MD1 knock‐out and wild‐type (WT) mice were subjected to uninephrectomy and continuous saline or d‐aldosterone infusion and given 1% sodium chloride drinking water for 4 weeks. Echocardiographic and haemodynamic measurements, electrophysiological studies, Masson staining, and molecular analysis were performed. Aldosterone‐infused WT mice develop HFpEF with left ventricular hypertrophy, moderate hypertension, pulmonary congestion, and diastolic dysfunction. Aldosterone infusion increased the vulnerability of WT mice to AF, as shown by a prolonged interatrial conduction time, shortened effective refractory period, and higher incidence of AF. In addition, aldosterone infusion increased myocardial fibrosis and inflammation, decreased sarcoplasmic reticulum Ca^2+^‐ATPase 2a protein expression and the phosphorylation of phospholamban at Thr17, and increased sodium/calcium exchanger 1 protein expression and the phosphorylation of ryanodine receptor 2 in WT mice. All of the above adverse effects of aldosterone infusion were further exacerbated in MD1 knock‐out mice compare with WT mice. Mechanistically, MD1 deletion increased the activation of the toll‐like receptor 4/calmodulin‐dependent protein kinase II signalling pathway in *in vivo* and *in vitro* experiments.

**Conclusions:**

MD1 deficiency increases the vulnerability of HFpEF mice to AF. This is mainly caused by aggravated maladaptive left atrial fibrosis and inflammation and worsened dysregulation of calcium handling, which is induced by the enhanced activation of the toll‐like receptor 4/calmodulin‐dependent protein kinase II signalling pathway.

## Introduction

Heart failure (HF) with presented ejection fraction (HFpEF) has a high prevalence and poor prognosis.^1^, ^2^ Clinical trials and registries have shown that atrial fibrillation (AF) is highly prevalent in HFpEF, and patients with HFpEF are at increased risk of developing AF.^3^, ^4^, ^5^ Independent of several common risk factors, such as diabetes, obstructive sleep apnoea, smoking, hypertension, and obesity, there appear to be several mechanisms that drive HFpEF patients to develop AF.^6^ It is vital to note that much of the current understanding of the mechanisms behind AF in patients with HFpEF has been derived from experiment models of HF with reduced EF (HFrEF). Unlike HFrEF, in which multiple therapeutic agents have been shown to improve survival, to date, all drugs and devices that have been tested rigorously have failed in patients with HFpEF.^7^, ^8^ Therefore, there is currently a need for experimental animal models of HFpEF population and identify valid therapeutic targets for improving the prognosis of HFpEF and preventing the occurrence of AF.

Previous experimental models of HF have also demonstrated that calcium handling disorder plays a critical role in arrhythmogenic mechanisms for the initiation of AF. The dysregulation of calcium handling in the failing heart can increase the ectopic triggered activity of atrial myocytes.^9^, ^10^ Calmodulin‐dependent protein kinase II (CaMKII), a pro‐arrhythmic signalling molecule, is critically involved in calcium handling disorder, in which altered sarcoplasmic reticulum (SR) Ca^2+^ handling proteins contribute to enhanced SR Ca^2+^ leak and AF development.^11^, ^12^, ^13^ In addition, a recent study found that the toll‐like receptor (TLR4)/CaMKII signalling pathway can affect calcium homoeostasis in atrial myocytes and increase the susceptibility of high fat‐diet (HFD)‐fed mice to AF.^14^

Myeloid differentiation protein 1 (MD1) is a type of secreted glycoprotein that can form a complex with radioprotective protein 105 (RP105) called MD1‐RP105.^15^ The MD1‐RP105 complex can directly interact with the MD2‐TLR4 complex by lateral binding, acting as a negative physiological regulator of the TLR4 signalling pathway.^16^ Our previous study showed that MD1 is widely expressed in the heart, and MD1 deletion leads to a more pronounced activation of the TLR4/CaMKII signalling pathway, which may further significantly influence the expression levels of Ca^2+^ handling proteins, increasing the vulnerability of HF mice to ventricular arrhythmia.^17^ Therefore, we hypothesized that MD1 deletion can alter calcium homoeostasis in atrial myocytes and increase susceptibility to AF in HFpEF by enhancing the activation of TLR4/CaMKII signalling.

To further investigate this possibility, we used loss‐of‐function approaches and observed that MD1 deletion can aggravate maladaptive left atrial (LA) fibrosis and inflammation, deteriorate dysregulation of calcium handling, and increase the vulnerability of HFpEF mice to AF. Mechanistically, MD1 deletion markedly enhances the activation of the TLR4/CaMKII signalling pathway.

## Methods

An expanded Methods section describing all procedures and protocols can be found in the Supporting Information.

### Myeloid differentiation protein 1 knock‐out mice

Myeloid differentiation protein 1 knock‐out (MD1‐KO) mice were generated as described in previous studies.^17^, ^18^ In brief, MD1‐KO mice were purchased from the Japan RIKEN BioResource Centre Mouse (BRC) (B6.129P2‐MD‐1 < tm1Kmiy>). The deletion of MD1 was confirmed in the obtained MD1‐KO mice by western blot analysis of LA tissues.

### Experimental Model

The use of all experimental mice were approved by the Animal Care and Use Committee of Renmin Hospital of Wuhan University and was consistent with the *Guide for the Care and Use of Laboratory Animals* published by the US National Institutes of Health (the eighth edition, National Resource Center 2011). The HFpEF mice underwent uninephrectomy and aldosterone infusion as previously described.^19^, ^20^ Briefly, 8‐week‐old MD1‐KO mice and wild‐type (WT) littermates were anaesthetized with pentobarbital sodium (50 mg/kg, intraperitoneally). They were subjected to uninephrectomy and intraperitoneal implantation of osmotic mini‐pumps (Durect Corp, Cupertino, CA) that delivered a continuous infusion of either saline or 0.15‐μg/h aldosterone (IA0700, Solarbio Co., China) for 4 weeks, accompanied by 1% sodium chloride (NaCl) intake. The four groups studied were as follows: (i) the WT‐Sal group (*n* = 20), WT mice infused with saline; (ii) the KO‐Sal group (*n* = 21), MD1‐KO mice infused with saline; (iii) the WT‐Aldo group (*n* = 20), WT mice infused with aldosterone; and (iv) the KO‐Aldo group (*n* = 21), MD1‐KO mice infused with aldosterone.

### Statistical analysis

Statistical analysis was performed using GraphPad Prism 5.0 (GraphPad, San Diego, CA). Continuous variables are shown as the means ± standard error of the mean. Statistical comparisons among multiple groups were performed with one‐way analysis of variance followed by Tukey's post hoc test and AF incidence across groups was compared using Fisher exact test. A value of *P* < 0.05 was considered statistically significant.

## Results

### The characteristics of the heart failure with preserved ejection fraction mouse model

None of the mice died during the 4 weeks of experiment. The characteristics of WT and KO mice 4 weeks after saline or aldosterone infusion are summarized in *Table*s 1 and *2*. First, aldosterone infusion significantly increased the heart weight/body weight (BW) of WT‐Aldo and KO‐Aldo mice. There were no significant differences in cardiac hypertrophy between WT‐Sham and KO‐Sham mice. However, cardiac hypertrophy in KO‐Aldo mice was significantly higher than that in WT‐Aldo mice. There was no difference in LA chamber size or left ventricular ejection fraction (LVEF) between WT and KO mice regardless of saline or aldosterone infusion. Second, haemodynamics revealed that the end‐systolic pressure and end‐diastolic pressure (EDP) were significantly increased after 4 weeks of aldosterone infusion in WT‐Aldo (125.08 ± 1.56 and 7.91 ± 0.4; *Table* 2) and KO‐Aldo mice (127.1 ± 2.32 and 10.4 ± 0.56; *Table 2*) compared with their respective saline‐infused controls. Third, aldosterone infusion significantly increased the lung weight (LW)/BW and LW/tibial length (TL), the indicator of pulmonary congestion, of WT‐Aldo mice vs. WT‐Sham mice (*P* < 0.05) and KO‐Aldo mice vs. KO‐Sham mice (*P* < 0.05; *Table* 1). There were no differences in the LW/BW and LW/TL between saline‐infused WT and KO mice. However, the LW/TL of KO‐Aldo mice were markedly higher than that of WT‐Aldo mice, indicating more pulmonary congestion (*P* < 0.05; *Table* 1). In addition, haemodynamics revealed diastolic dysfunction with increased LVEDP (dp/dt min) and LV pressure isovolumetric relaxation constant (tau) in Aldo mice vs. sham mice at 4 weeks (*Table* 2). Finally, serum aldosterone levels in WT‐Aldo and KO‐Aldo mice were markedly elevated compared with those in the saline‐infused mice. There was no difference in serum aldosterone levels between WT and KO mice regardless of saline or aldosterone infusion.

**Table 1:** Characteristics of wild‐type or myeloid differentiation protein 1 knock‐out mice 4 Weeks after saline/aldosterone infusion

Groups	WT‐Saline (*n* = 8)	KO‐Saline (*n* = 8)	WT‐Aldo (*n* = 8)	KO‐Aldo (*n* = 8)
BW (g)	21.56 ± 0.35	22.28 ± 0.31	22.81 ± 0.34	23.59 ± 0.44^*^
HW/BW (mg/g)	4.56 ± 0.06	4.48 ± 0.07	5.82 ± 0.09^*^	6.82 ± 0.16^*,**^
LW/BW (mg/g)	4.98 ± 0.06	5.08 ± 0.1	6.96 ± 0.17^*^	7.77 ± 0.21^*,**^
LW/TL (mg/g)	5.85 ± 0.07	6.09 ± 0.1	8.5 ± 0.12^*^	9.94 ± 0.15^*,**^
LVEDd (mm)	3.9 ± 0.08	3.83 ± 0.09	3.85 ± 0.1	3.96 ± 0.07
LVESd (mm)	2.18 ± 0.06	2.11 ± 0.07	2.15 ± 0.07	2.35 ± 0.13
LVEF (%)	81.63 ± 0.8	82.12 ± 0.95	80.13 ± 1.91	77.13 ± 2.95
LVFS (%)	44.38 ± 0.01	44.63 ± 0.01	43.88 ± 0.02	40.63 ± 0.03
LAD (mm)	1.55 ± 0.04	1.59 ± 0.04	1.56 ± 0.1	1.59 ± 0.07
Serum aldosterone levels (pg/mL)	235.3 ± 19.09	238.79 ± 15.13	438.47 ± 12.85^*^	458.48 ± 10.8^*^

Aldo, Aldosterone; BW, body weight; HW, heart weight; KO, knock‐out; LAD, left atrial diameter; LVEDd, left ventricle end‐diastolic dimension; LVEF, left ventricular ejection fraction; LVFS, left ventricular fractional shortening; LVESd, left ventricular end‐systolic diameter; LW, lung weight; TL, tibial length.

Data are expressed as Mean ± standard error of the mean.

* *P* < 0.05 vs. WT‐Sal group.

** *P* < 0.05 vs. WT‐Aldo group.

**Table 2:** Invasive haemodynamic measurements

Parameters	WT‐Sham (*n* = 6)	KO‐Sham (*n* = 6)	WT‐Aldo (*n* = 6)	KO‐Aldo (*n* = 6)
Pressure volume measurements				
HR (bpm)	479.91 ± 16.79	487.18 ± 22.53	468 ± 19.17	451.15 ± 11.3
LVESP (mmHg)	114.38 ± 2.28	113.18 ± 2.09	125.08 ± 1.56^*^	127.1 ± 2.32^*^
LVEDP (mmHg)	6.16 ± 0.48	6.12 ± 0.69	7.91 ± 0.4^*^	10.4 ± 0.56^*,**^
LVESV (uL)	28.13 ± 0.62	27.82 ± 0.72	25.26 ± 0.83^*^	25.22 ± 0.76^*^
LVEDV (uL)	45.02 ± 0.74	45.22 ± 1.16	40.8 ± 1.04^*^	40.45 ± 0.66^*^
SV (uL)	20.29 ± 0.78	19.74 ± 0.54	18.32 ± 0.72^*^	17.57 ± 0.84^*,**^
CO (mL/min)	10 378 ± 255.71	9964.25 ± 306.88	8277.63 ± 286.63^*^	7349.63 ± 300.32^*,**^
Ea (mmHg/uL)	5.82 ± 0.24	5.93 ± 0.27	7.06 ± 0.27^*^	8.05 ± 0.18^*,**^
Systolic function				
EF (%)	43.15 ± 0.98	42.74 ± 1.59	43.8 ± 1.32	40.98 ± 1.38
SW (mmHg^*^uL)	1881 ± 96.38	1737.88 ± 76.67	1766.5 ± 75.19	1666.13 ± 52.61
dp/dt max (mmHg/s)	14 182.75 ± 678.67	14 441.25 ± 703.76	13 833.75 ± 702.75	12 286.25 ± 356.47^*^
Diastolic function				
TAU (ms)	6 ± 0.34	6.27 ± 0.33	8.59 ± 0.22^*^	10.51 ± 0.49^*,**^
dp/dt min (mmHg/s)	−10625.75 ± 305.95	−11497.75 ± 697.78	−9057.25 ± 200.11^*^	−7546 ± 321.78^*,**^

CO, cardiac output; dp/dt max, maximum rate of increase in left ventricular pressure; dp/dt min, maximum rate of decrease in left ventricular pressure; Ea, arterial elastance (measure of ventricular afterload); EF, ejection fraction; HR, heart rate; LVEDP, left ventricular end‐diastolic pressure; LVEDV, left ventricular end‐diastolic volume; LVESP, left ventricular end‐systolic pressure; LVESV, left ventricular end‐systolic volume; SV, stroke volume; SW, stroke work; TAU, left ventricular isovolumetric relaxation constant (relaxation time constant calculated by the Weiss method).

Data express as Mean ± SEM.

* *P* < 0.05 vs. WT‐Sal group.

** *P* < 0.05 vs. WT‐Aldo group.

Therefore, all of the above data showed that mice subjected to uninephrectomy and aldosterone infusion for 4 weeks developed HFpEF with LV hypertrophy, moderate hypertension, pulmonary congestion, and diastolic dysfunction while maintaining a normal/preserved LVEF, and the above adverse effects were further exacerbated by MD1 deletion.

### Myeloid differentiation protein 1 deletion increased the vulnerability of heart failure with preserved ejection fraction mice to atrial fibrillation

**Figure 1:**
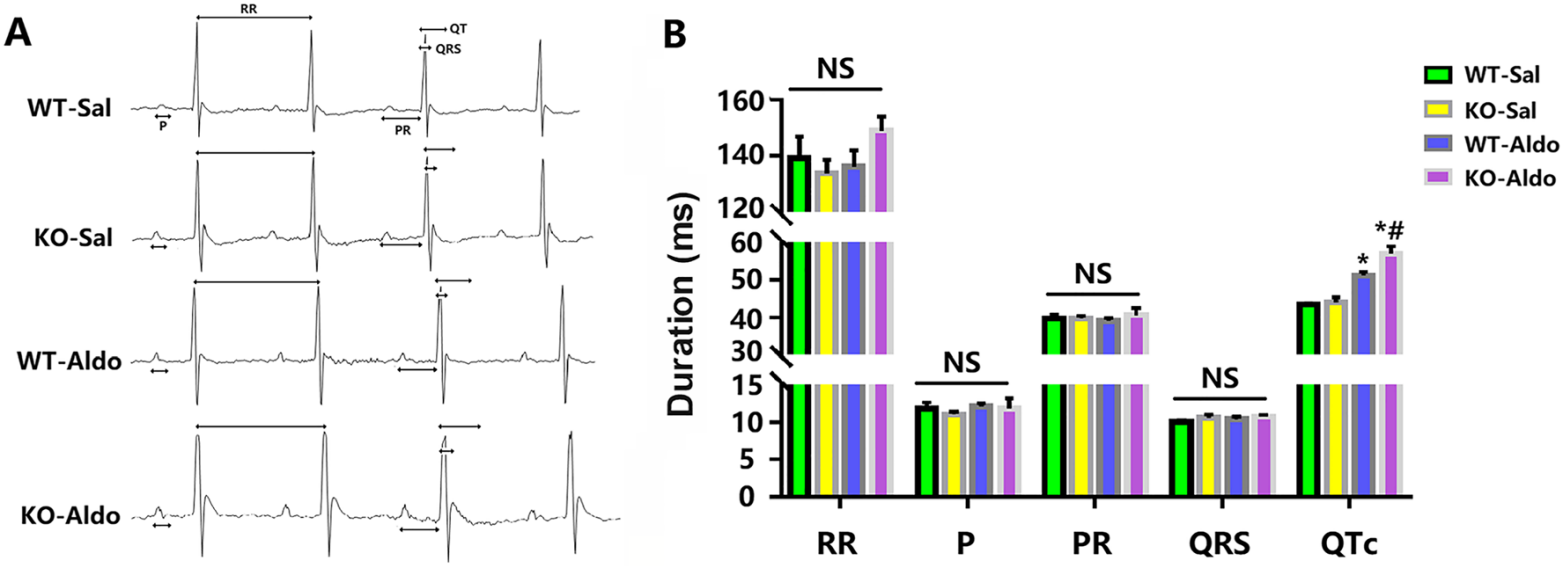
Analysis of surface electrocardiograph in wide‐type (WT) and myeloid differentiation protein 1 knock‐out mice 4 weeks after saline or aldosterone infusion. (A) A representative trace of the surface electrocardiography. RR interval, P wave duration, PR interval, QRS duration, and QTc interval were indicated as an arrow. (B) Surface electrocardiography parameters (*n* = 8). Data are expressed as mean ± standard error of the mean. ^*^ *P* < 0.05 vs. WT‐Sal group and ^#^ *P* < 0.05 vs. WT‐Aldo group.

To clarify the underlying role of MD1 in susceptibility to HFpEF‐related AF, we first monitored electrical signals by surface electrocardiogram. There was no difference in RR intervals, P waves, PR intervals, or QRS durations between the WT and KO mice regardless of saline or aldosterone infusion (*Figure* 1 *A, B*). However, aldosterone infusion significantly increased QTc intervals in the WT‐Aldo and KO‐Aldo mice. There was no significant difference in QTc intervals between the WT‐Sal and KO‐Sal mice (*Figures* *1* *A* and *1* *B*).

**Figure 2:**
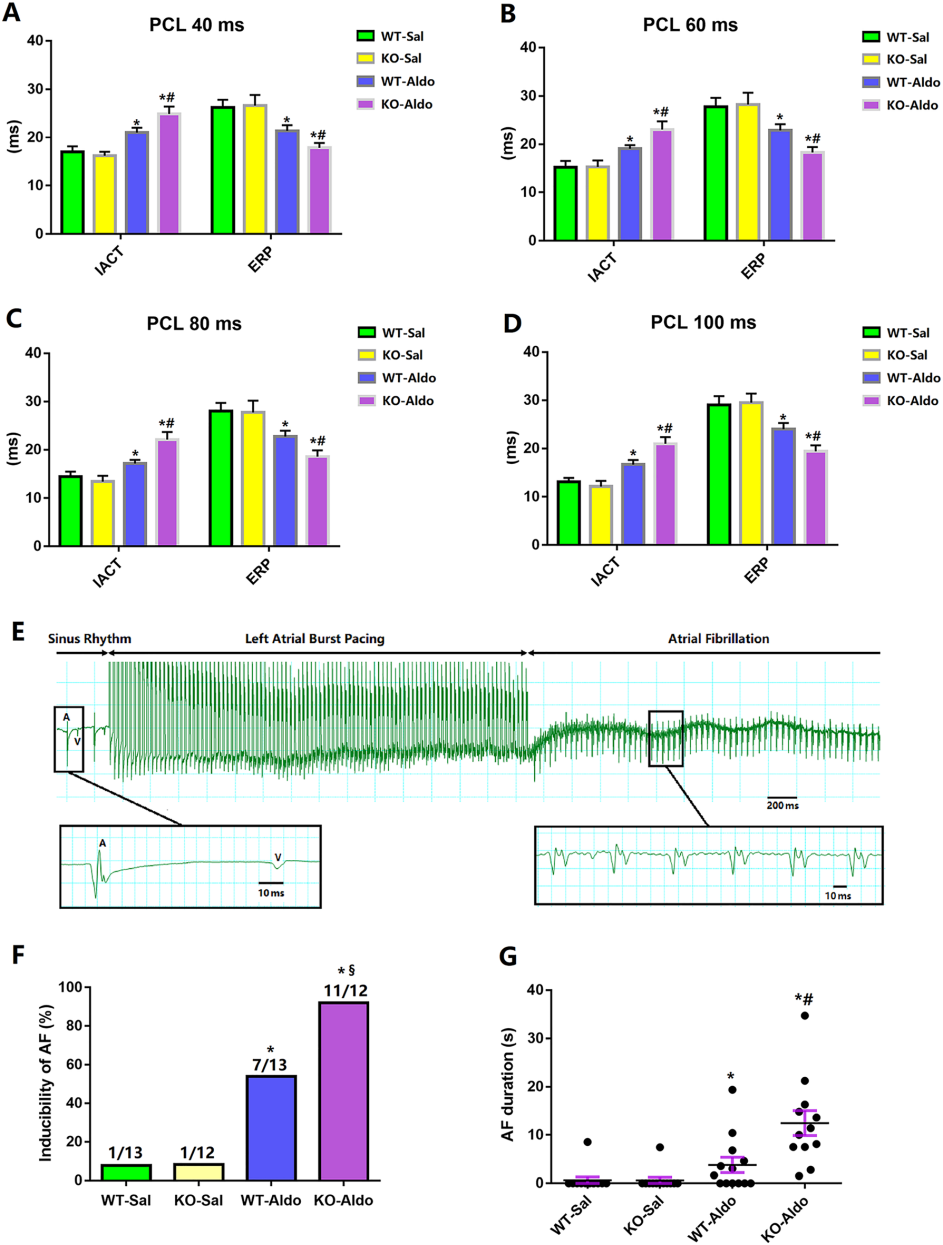
The electrophysiological properties of the atrial in wide‐type (WT) and myeloid differentiation protein 1 knock‐out mice 4 weeks after saline or aldosterone infusion. (A–D) Intra‐atrial conduction time (IACT) and effective refractory period (ERP) of the left atrium evaluated in the study using isolated perfused heart mice (*n* = 8). (E) Representative electrograms of atrial fibrillation (AF) induction after atrial burst pacing in the isolated perfused heart using Langendorff apparatus in a knock‐out (KO)‐Aldo mouse. (F,G) Inducibility and duration of AF in the study using isolated perfused heart (*n* = 12–13). Data are expressed as mean ± standard error of the mean. ^*^ *P* < 0.05 vs. wild‐type (WT)‐Sal group, ^#^ *P* < 0.05 vs. WT‐Aldo group, and ^§^ *P* < 0.1 vs. WT‐Aldo group.

In addition, we further characterized changes in electrophysiological parameters [the atrial effective refractory period (ERP), intra‐atrial conduction time (IACT), and incidence of AF] in Langendorf‐perfused hearts. Aldosterone infusion significantly prolonged the IACT of 40, 60, 80, and 100‐ms cycles in WT‐Aldo mice vs. WT‐Sal mice (*P* < 0.05) and KO‐Aldo mice vs. KO‐Sal mice (*P* < 0.05). The increase in the IACT in KO‐Aldo mice was significantly attenuated in WT‐Aldo mice (*Figure*s 2 *A*–2 *D*). Moreover, aldosterone infusion significantly decreased the LA ERP of 40, 60, 80, 100‐ms basic cycles tested in WT‐Aldo mice vs. WT‐Sal mice (*P* < 0.05) and KO‐Aldo mice vs. KO‐Sal mice (*P* < 0.05). The shortening of the IACT in KO‐Aldo mice was significantly attenuated in WT‐Aldo mice (*Figure*s 2 *A*–*D*). Moreover, aldosterone infusion significantly increased the AF induction rate in WT‐Aldo mice vs. WT‐Sal mice (*P* < 0.05) and KO‐Aldo mice vs. KO‐Sal mice (*P* < 0.05). The increase in the AF induction rate in WT‐Aldo mice (7/13, 53.8%) was significantly decreased compared with KO‐Aldo mice (11/12, 91.7%) (*Figure* 2 *F*). These results indicated that loss of MD1 increased the vulnerability of HFpEF mice to AF.

### Myeloid differentiation protein 1 knock‐out aggravated left atrial fibrosis in heart failure with preserved ejection fraction mice

**Figure 3:**
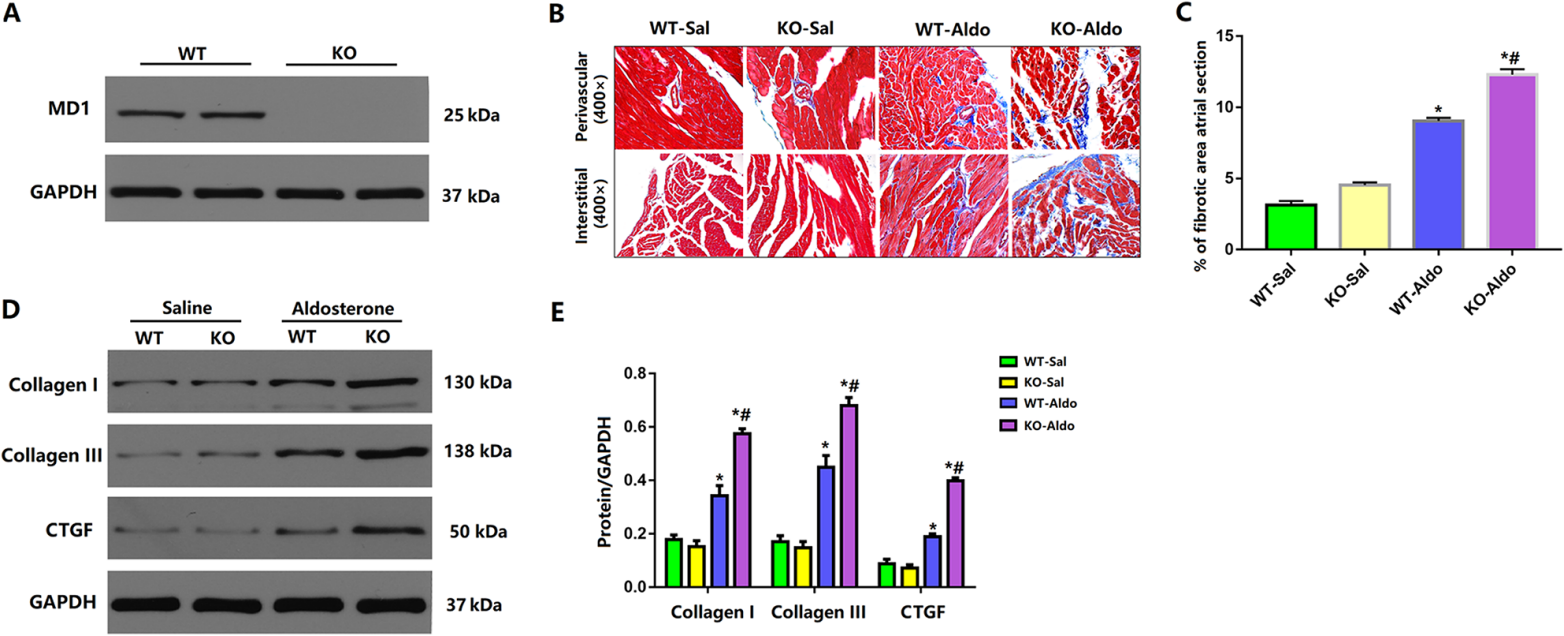
Myocardial fibrosis in wild‐type (WT) and Myeloid differentiation protein 1 (MD1)‐knock‐out mice 4 weeks after saline or aldosterone infusion. (A) Representative western blots of MD1 expression in left atrial tissues from WT and MD1‐KO mice (*n* = 6). (B) Representative Masson trichrome staining. Original magnification ×400; (C) myocardial fibrosis area (*n* = 4); and (D,E) representative western blots and statistical analysis of the fibrosis‐related proteins levels (*n* = 4). Data are expressed as mean ± standard error of the mean. ^*^ *P* < 0.05 vs. WT‐Sal group and ^#^ *P* < 0.05 vs. WT‐Aldo group.

Atrial fibrosis is a common process by which HFpEF promotes AF, which ultimately creates heterogeneity of conduction within the atria and thus acts as a substrate for re‐entry.^21^, ^22^ Therefore, we also detected the state of fibrosis in four groups. Western blot analysis of LA tissue samples indicated successful knock‐out of MD1 in WT‐KO mice (*Figure* 3 *A*). Aldosterone infusion significantly increased the area of myocardial fibrosis in WT‐Aldo mice vs. WT‐Sal mice (*P* < 0.05) and KO‐Aldo mice vs. KO‐Sal mice (*P* < 0.05; *Figures* *3* *B* and *3* *C*). Myocardial fibrosis in KO‐Aldo mice was significantly increased compared with that in WT‐Aldo mice (*P* < 0.05; *Figures* *3* *B* and *3* *C*). Consistent with these findings, the mRNA expression of molecular markers (collagen I, collagen III, and transforming growth factor‐β1) of cardiomyocyte fibrosis was increased in the LA of WT‐Aldo mice vs. WT‐Sal mice (*P* < 0.05) and KO‐Aldo mice vs. KO‐Sal mice (*P* < 0.05; *Figures* *3* *C* and *3* *D*).

### Myeloid differentiation protein 1 knock‐out aggravated left atrial inflammation in heart failure with preserved ejection fraction mice

**Figure 4:**
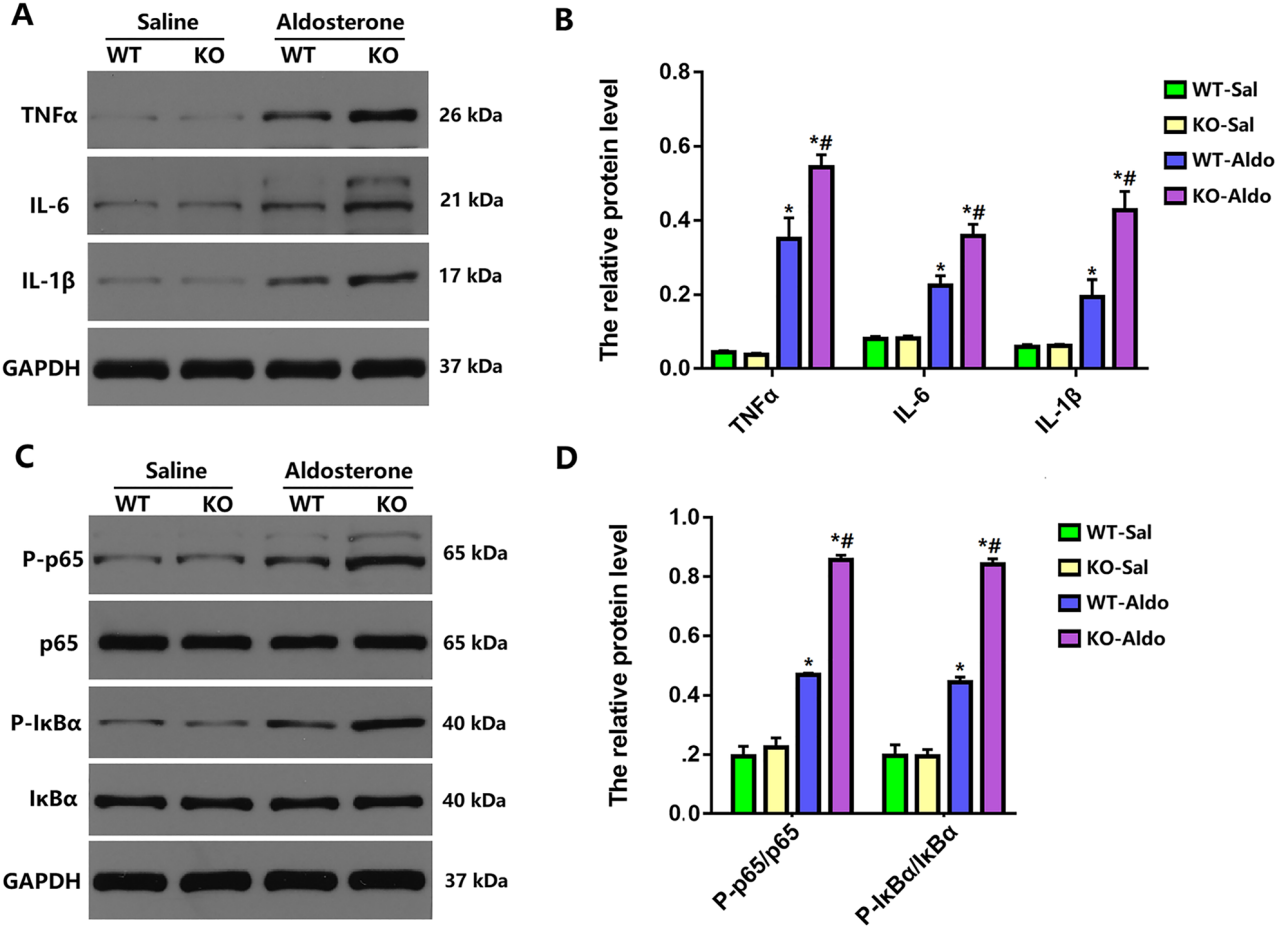
Myocardial inflammation in wild‐type (WT) and myeloid differentiation protein 1 knock‐out mice 4 weeks after saline or aldosterone infusion. (A,B) Representative western blots and statistical analysis of the inflammation‐related protein levels (*n* = 4). (C,D) Representative western blots and statistical analysis of p65, P‐p65, inhibitor of kappa B (IκBα), and P‐IκBα (*n* = 4). Data are expressed as mean ± standard error of the mean. ^*^ *P* < 0.05 vs. WT‐Sal group and ^#^ *P* < 0.05 vs. WT‐Aldo group.

Inflammation is the most crucial pathological response to cardiac damage and repair.^23^ Moreover, there is extensive evidence that inflammation contributes to the pathophysiology of AF.^24^ Therefore, we also explored the expression levels of cytokines secreted by inflammatory cells. The mRNA levels of the proinflammatory cytokines interleukin (IL)‐1β, IL‐6, and tumour necrosis factor (TNF)‐α were increased in the LA of WT‐Aldo mice vs. WT‐Sal mice (*P* < 0.05) and KO‐Aldo mice vs. KO‐Sal mice (*P* < 0.05). The increase in proinflammatory cytokines in KO‐Aldo mice was markedly attenuated in WT‐Aldo mice (*P* < 0.05) (*Figure* 4 *A*). Our previous study reported that the nuclear factor kappa B (NF‐κB) signalling pathway play a vital role in regulating the inflammatory response and that MD1 has an effect on NF‐κB suppression in the setting of obesity.^18^ Therefore, the NF‐κB signalling pathway was investigated to confirm MD1 function after aldosterone infusion. As shown in *Figure* 4 *B*, the levels of phosphorylated p65 and inhibitor of kappa B (IκBα) were substantially increased in KO‐Aldo mice compared with WT‐Aldo mice, indicating that the NF‐κB signalling pathway was strongly activated by MD1 deletion. These data suggested that MD1 modulates atrial inflammation by interfering with the NF‐κB signalling pathway to some extent.

### Loss of myeloid differentiation protein 1 knock‐out worsened the dysregulation of calcium handling in heart failure with preserved ejection fraction mice

Extensive studies have suggested that alterations in calcium‐handling proteins, including ryanodine receptor 2 (RyR2), SR Ca^2+^‐ATPase 2a (SERCA2a), phospholamban (PLB), and sodium/calcium exchanger 1 (NCX1), contribute to changes in intracellular calcium transients and diastolic SR Ca^2+^ release that, in turn, lead to Ca^2+^‐triggered arrhythmogenesis in HF animal models.^9^, ^25^, ^26^, ^27^ Therefore, we next detected the protein expression of RyR2, SERCA2a, PLB, and NCX1 in each group.

**Figure 5:**
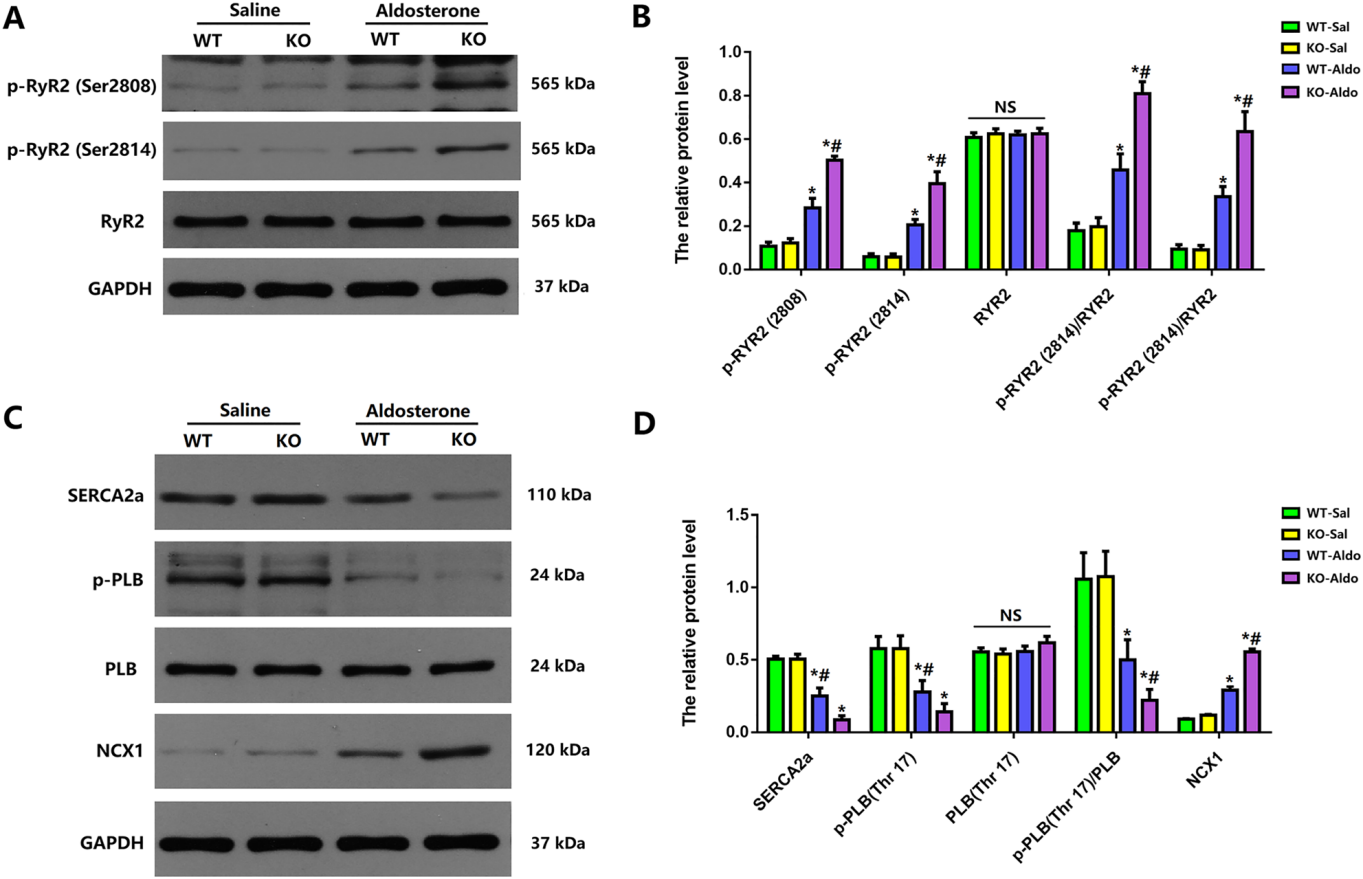
Expression of calcium handling regulatory proteins in wild‐type (WT) and and myeloid differentiation protein 1 knock‐out mice 4 weeks after saline or aldosterone infusion. (A,B) Representative western blots and statistical analysis of ryanodine receptor 2 (RyR2) phosphorylation at Ser2814 and RyR2 phosphorylation at Ser2808, RyR2, and GAPDH (*n* = 4). (C,D) Representative western blots and statistical analysis of sarcoplasmic reticulum Ca^2+^‐ATPase 2a (SERCA2a) and phospholamban (PLB) phosphorylation at Thr17, PLB, sodium/calcium exchanger (NCX), and GAPDH (n = 4). Data are expressed as mean ± standard error of the mean. ^*^ *P* < 0.05 vs. WT‐Sal group and ^#^ *P* < 0.05 vs. WT‐Aldo group.

First, the phosphorylation of RyR2 at Ser 2808 and Ser 2814 was markedly increased by aldosterone infusion in both WT and MD1‐KO hearts, and the adverse effect of phosphorylation at Ser 2808 and Ser 2814 was further aggravated in KO‐Aldo mice compared with WT‐Aldo mice (*Figures* *5* *A* and *5* *B*). However, there was no difference in RyR2 protein expression between WT and KO mice regardless of saline or aldosterone infusion.

In addition, aldosterone infusion significantly decreased the protein expression of SERCA2a and phosphorylated PLB (Thr17) in WT‐Aldo mice vs. WT‐Sal mice (*P* < 0.05) and KO‐Aldo mice vs. KO‐Sal mice (*P* < 0.05). The decrease in the protein expression of SERCA2a and phosphorylated PLB (Thr17) in KO‐Aldo mice was significantly attenuated in WT‐Aldo mice. However, there was no difference in PLB protein expression between WT and MD1‐KO mice regardless of saline or aldosterone infusion (*Figures* *5* *C* and *D*).

Finally, aldosterone infusion significantly increased NCX protein expression in WT‐Aldo mice vs. WT‐Sal mice (*P* < 0.05) and KO‐Aldo mice vs. KO‐Sal mice (*P* < 0.05). The increase in NCX1 protein expression in KO‐Aldo mice was markedly attenuated in WT‐Aldo mice (*Figures* *5* *C* and *5* *D*). Therefore, our data showed that the loss of MD1 worsened the dysregulation of SERCA2, the phosphorylation of RyR2 at Ser 2808 and Ser 2814, and the phosphorylation of PLB at Thr17 in HFpEF mice.

### Myeloid differentiation protein 1 knock‐out regulated the activation of the toll‐like receptor 4/calmodulin‐dependent protein kinase II signalling pathway *in vivo* and *in vitro*

**Figure 6:**
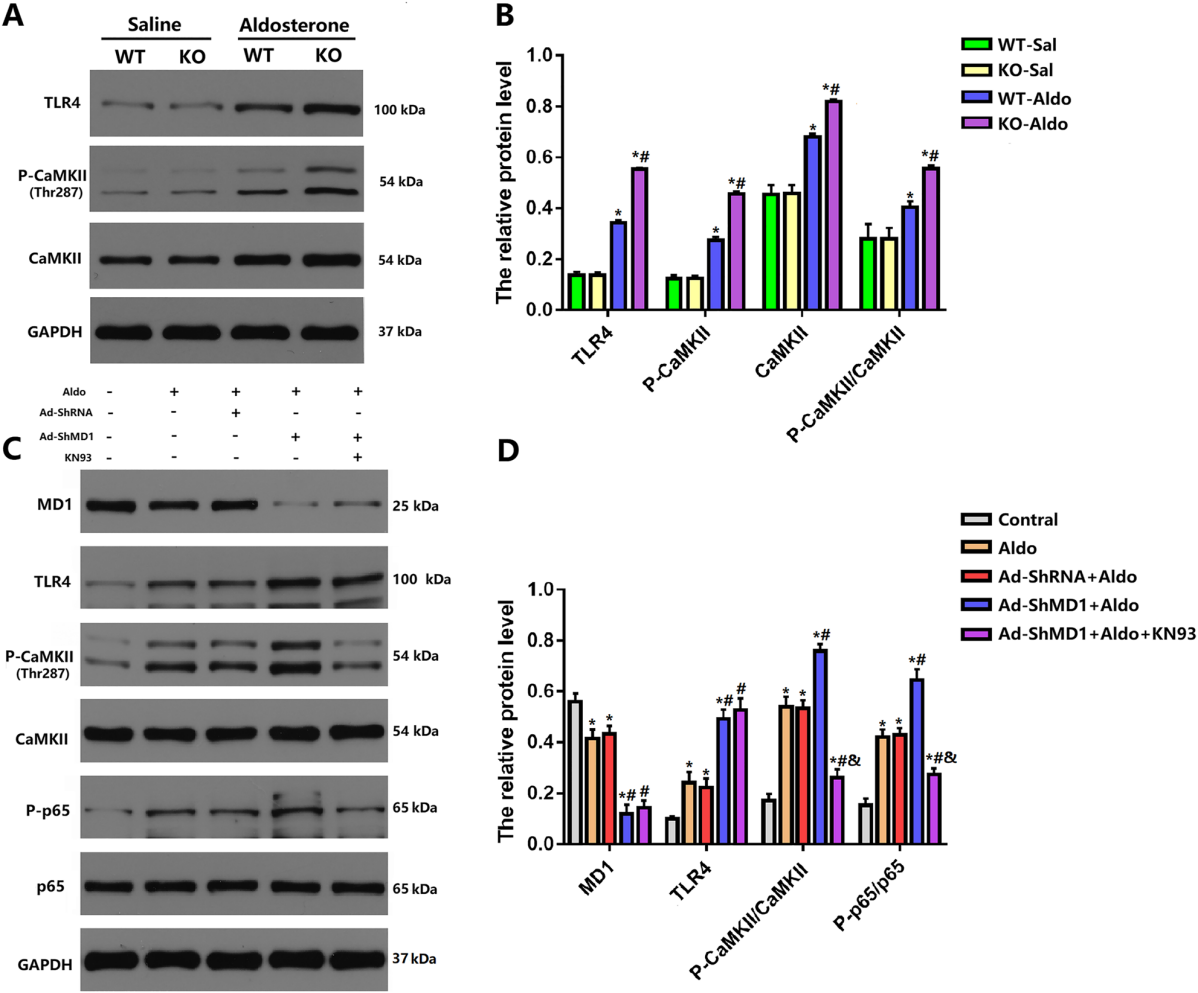
Myeloid differentiation protein 1 (MD1) regulates toll‐like receptor 4 (TLR4)/calmodulin‐dependent protein kinase II (CaMKII) signalling in *in vivo* and *in vitro* experiments. (A,B) Representative western blots and statistical analysis of TLR4, CaMKII, and P‐CaMKII in wild‐type (WT) and MD1‐knock‐out (KO) mice 4 weeks after saline or aldosterone infusion (*n* = 4). Data are expressed as mean ± standard error of the mean, ^*^ *P* < 0.05 vs. WT‐Sal group and ^#^ *P* < 0.05 vs. WT‐Aldo group. (C, D) Representative western blots and statistical analysis of MD1, TLR4, P‐CaMKII, CaMKII, P‐p65, and p65 in aldosterone‐induced H9C2 cell (*n* = 4). Data are expressed as mean ± standard error of the mean. ^*^ *P* < 0.05 vs. Control, ^#^ *P* < 0.05 vs. Aldo, and ^&^ *P* < 0.05 vs. Ad‐ShMD1.

The results above indicate that MD1‐KO HFpEF mice may have increased vulnerability to AF through facilitated fibrosis and inflammation and the dysregulation of calcium handling. However, the underlying mechanism by which MD1‐KO exerts its pro‐arrhythmia response is unclear. The TLR4/CaMKII signalling pathway has been reported to play an important role in atrial arrhythmia through regulating atrial fibrosis, inflammation, and the function of calcium‐handling proteins.^14^, ^28^ Besides, according to our previous studies, MD1 has a suppressive effect on TLR4/CaMKII signalling pathway.^17^, ^29^ Thus the TLR4/CaMKII signalling pathway was investigated to confirm the function of MD1. As shown in *Figures* *6* *A* and *6* *B*, the levels of TLR4 and phosphorylated CaMKII were substantially increased in KO‐Aldo mice, indicating that the TLR4/CaMKII signalling pathway was strongly activated by MD1 deletion. These data suggested that MD1 modulated atrial fibrosis and calcium‐handling proteins by affecting the TLR4/CaMKII signalling pathway.

To confirm the modulation of the TLR4/CaMKII signalling by MD1, the CaMKII inhibitor KN93 was used to pre‐treat H9c2 cells before aldosterone stimulation. H9C2 cells were infected with Ad‐shMD1 or Ad‐shRNA and then treated with 1‐μM aldosterone for 18 h.^30^, ^31^ Consistent with the *in vivo* results, the aldosterone‐induced activation of CaMKII and P65 was enhanced in MD1 knockdown cells. In addition, we exposed cultured H9C2 cells that had been previously infected with Ad‐shMD1 to a CaMKII inhibitor, KN93, for 30 min and then treated with aldosterone for 18 h. The protein phosphorylation levels of CaMKII and P65 were increased following aldosterone stimulation, and this was significantly suppressed by KN93 compared with the no treatment (*Figures* *6* *C* and *6* *D*). Therefore, the *in vitro* experiment further verified that the inactivation of the TLR4/CaMKII signalling pathway rescued the adverse effect of MD1 deficiency in aldosterone‐induced myocyte remodelling. In brief, all of the above data demonstrated that MD1 modulated the TLR4/CaMKII signalling pathway in aldosterone‐induced pathological myocyte remodelling.

## Discussion

In the present study, aldosterone‐infused WT mice developed HFpEF with LV hypertrophy, moderate hypertension, pulmonary congestion, and diastolic dysfunction while maintaining a preserved LVEF, and the above adverse effects were further exacerbated by MD1 deletion. Moreover, the loss of MD1 increased the vulnerability of aldosterone‐induced HFpEF mice to AF, as shown by the prolonged IACT, shortened EDP, and higher incidence of AF. In addition, aldosterone infusion markedly increased myocardial fibrosis and inflammation, decreased SERCA2a protein expression and the phosphorylation of PLB at Thr17, and increased NCX1 protein expression and the phosphorylation of RyR2 in MD1‐KO mice. Finally, MD1 deletion markedly enhanced the activation of the TLR4/CaMKII signalling pathway *in vivo* and *in vitro*. These findings suggest that MD1 may be a potential therapeutic target for reducing the incidence of AF in HFpEF patients.

An ideal animal model should meet various requirements for mimicking human disease, including cardiac, haemodynamic, neurohormonal, and peripheral aberrations commonly seen in HFpEF patients. A recent review^32^ comprehensively summarized current models of HFpEF and found that a mouse model of uninephrectomy, aldosterone infusion, and 1% NaCl administration recapitulates clinical HFpEF phenotypes and features molecular changes that have been described clinically. Several studies have consistently shown that the combination of uninephrectomy, aldosterone infusion, and 1% NaCl administration induces an experimental model that mimics HFpEF and can be used to study the pathogenesis of HFpEF *in vivo*.^31^, ^33^, ^34^ These studies showed that HFpEF mice exhibit moderate hypertension, LV hypertrophy, pulmonary congestion, and diastolic dysfunction while maintaining a preserved LVEF. Consistent with our observations, aldosterone‐infused WT and MD1‐KO mice showed cardiac hypertrophy, a higher end‐systolic pressure and EDP, increased LW/TL, lower dp/dt min, and preserved LVEF. The above adverse effects in WT‐Aldo mice were significantly decreased in KO‐Aldo mice. Therefore, the HFpEF mouse model was successfully established, and MD1‐KO mice exhibited worsened cardiac diastolic function than that of WT‐Aldo mice.

Clinical studies have shown that over 30% of patients with incident HFpEF have prevalent AF, and the presence of AF is more strongly linked to incident HFpEF than to HFrEF.^35^, ^36^ Consistent with these data, our study showed that the AF induction rate was significantly increased in aldosterone‐induced HFpEF mice. In addition, the IACT and LA ERP were markedly increased and decreased, respectively, in aldosterone‐induced HFpEF mice. All of the above changes were worse in KO‐Aldo mice. Several studies have shown that, before the onset of AF, shorter atrial ERPs and a longer IACT are associated with higher inducibility of AF in HF patients and animals.^37^, ^38^, ^39^ Moreover, earlier work from our group showed that MD1 deletion can increase the AF induction rate in the setting of HFD.^18^ Therefore, a deficiency in MD1 can increase vulnerability to AF caused by aldosterone‐induced HFpEF.

The importance of atrial fibrosis in the initiation and re‐entrance of AF, especially in the matter of conduction disturbances, is widely understood.^40^ Atrial interstitial fibrosis has been shown to increase AF vulnerability in animal models of HF.^22^, ^41^, ^42^ According to previous research, ERP shortening may be explained by an increase in the number of fibroblasts, which shortens the duration of the action potential.^43^ In the present study, aldosterone infusion significantly shortened the ERP and increased LA fibrosis in WT‐Aldo mice compared with the WT‐Sal mice. The shortened ERP and increased atrial fibrosis in KO‐Aldo mice were substantially worse than those in WT‐Aldo mice. According to the findings of this study, loss of MD1 exacerbated atrial fibrosis and increased the vulnerability of aldosterone‐induced HFpEF mice to AF.

Human and animal studies have demonstrated that inflammation plays an essential role in the pathophysiology of HFpEF.^44^, ^45^, ^46^ Many studies have shown that inflammation plays a vital role in triggering AF. Li *et al*. observed that TNF‐α blood levels were higher in patients with AF compared with those in sinus rhythm and in persistent and permanent AF compared with paroxysmal AF.^47^ Moreover, a recent study also suggested that higher IL‐6 blood levels are associated with greater AF risk in the general population.^48^ Consistent with this possibility, our present correlative evidence suggested that chronic aldosterone infusion increased the levels of proinflammatory cytokines IL‐1β, IL‐6, and TNF‐α and vulnerability to AF. Besides, in our study, chronic aldosterone infusion substantially increased the levels of phosphorylated p65 and IκBα in KO‐Aldo mice compared with WT‐Aldo mice, indicating that the NF‐κB pathway was markedly activated by MD1 deletion. Supporting these data, our previous study has shown that MD1 deficiency increases the vulnerability HFD‐fed mice to AF, mainly due to an increase in the proinflammatory IL‐1β, IL‐6, and TNF‐α, a decrease in the levels of the anti‐inflammatory cytokine IL‐10, and the facilitation of atrial fibrosis. This mainly occurs through an increase in the levels of phosphorylated p65 and IκBα because of the enhanced activation of the TLR4 signalling pathway.^18^ These observations are consistent with a recent review by Queisser and Schupp showing that aldosterone can activate the NF‐κB pathway in humans and different experimental models.^49^ Some studies have reported that NF‐κB can regulate the expression of sodium channel subunits that contribute to AF‐associated electrical remodelling.^50^ Although ion channels were not investigated in this study, we are confident that the loss of MD1 increases AF susceptibility by regulating cardiac inflammation.

Extensive studies have suggested that alterations in Ca^2+^ handling proteins contribute to changes in intracellular Ca^2+^ transients and diastolic SR Ca^2+^ release that, in turn, lead to Ca^2+^‐triggered atrial arrhythmogenesis.^11^, ^12^, ^25^, ^51^ Recent studies have indicated that alterations in CaMKII‐dependent RyR2 phosphorylation are also exhibited in the atrium of chronic AF patients.^11^, ^13^ Abnormal diastolic RyR2 Ca^2+^ release may be the primary cause of abnormal Ca^2+^ handling in HF and chronic AF.^9^, ^26^, ^27^ In addition, increased diastolic SR Ca^2+^ leakage along with impaired function of Ca^2+^ uptake because of lower expression of the SERCA inhibitory protein PLB in HF and increased Na influx via NCX for Ca^2+^ removal can abnormally trigger activity and initiate atrial arrhythmias.^52^, ^53^ In our study, chronic aldosterone infusion decreased SERCA2a protein expression and the phosphorylation of PLB at Thr17 and increased NCX1 protein expression and the phosphorylation of RyR2 but did not alter RyR2 or PLB protein expression. Phosphorylation of PLB at Thr17 site is a specific target of CaMKII, and activated CaMKII is associated with increased phosphorylation of PLB at Thr17 site.^54^ However, in our study, the level of phosphorylation of PLB at Thr17 is markedly decreased in the aldosterone‐induced heart, where activated CaMKII was observed, although no apparent change in total PLB abundance. Interestingly, this decrease occurred despite an increase in CaMKII activity characteristic of HF. The observed differences in p‐PLB could be because of the balance in protein phosphatase and/or protein phosphatase inhibitor. It has been proposed that in human failing myocardium, phosphorylation of Thr17 decreased because of increased activity of protein phosphatase 2B.^55^ All of the above changes lead to Ca^2+^‐triggered atrial arrhythmogenesis. In addition, alterations in Ca^2+^ handling proteins in WT‐Aldo mice were significantly worsened in KO‐Aldo mice. A recent study indicated that MD1‐KO can interfere with the expression of Ca^2+^ handling proteins in pressure‐induced HF mice.^17^ Therefore, there is strong and growing evidence that MD1 deletion worsens the dysregulation of calcium handling and increases the vulnerability of aldosterone‐induced HFpEF mice to AF.

The underlying mechanisms by which MD1 regulates the vulnerability of aldosterone‐induced HFpEF mice to AF may be associated with its downstream TLR4/CaMKII signalling pathway. Several studies have consistently shown that activated CaMKII can increase the phosphorylation of RyR2 and induce an imbalance between NCX1 and SERCA2, which lead to a disturbance in intracellular Ca^2+^ homoeostasis‐triggered activity and arrhythmia initiation in the setting of HF.^27^, ^56^, ^57^, ^58^ Furthermore, a recent study found that inhibiting CaMKII can attenuate atrial fibrosis, which creates heterogeneity of conduction within the atria and thus acts as a substrate for re‐entry.^21^, ^22^, ^59^ Additionally, the activation of CaMKII in cardiomyocytes triggers the activation of NF‐κB and the expression of inflammatory chemokines and cytokines and ultimately increased AF vulnerability.^60^, ^61^ Several studies have demonstrated that CaMKII can regulate NF‐κB activation under pathological conditions, such as myocardial infarction, ischaemia/reperfusion injury, and obesity, which suggests that CaMKII serves to trigger and sustain subsequent changes in inflammatory gene expression that contribute to cardiac stress.^28^, ^62^, ^63^ Our previous studies showed that MD1 deletion leads to a more pronounced activation of the TLR4/CaMKII signalling pathway, which may further significantly influence the expression levels of Ca^2+^ handling proteins, increasing the vulnerability of HF mice to ventricular arrhythmia.^14^ Consistent with our current research, the protein expression levels of P‐CaMKII/CaMKII were markedly increased in LA tissues from KO‐Aldo mice compared with WT‐Aldo mice, indicating that the TLR4/CaMKII signalling pathway was strongly activated by MD1 deletion. In addition, the *in vitro* experiment further verified that the inactivation of the TLR4/CaMKII signalling pathway rescued the adverse effect of MD1 deficiency by decreasing the phosphorylation levels of CaMKII and p65 in aldosterone‐induced myocyte remodelling. Therefore, we believe that the loss of MD1 enhanced the activation of the TLR4/CaMKII signalling pathway to facilitate cardiomyocyte arrhythmogenesis following aldosterone‐induced HFpEF.

### Novelty and limitations

We propose for the first time that the loss of MD1 can increase the vulnerability of a mouse model of HFpEF to AF and that MD1 may represent a novel therapeutic target for the treatment of HFpEF‐related remodelling of the atrium. However, the current study has some limitations. First, clinical and experimental evidences have been used to postulate the underlying complex pathophysiological mechanisms of AF, including electrical remodelling, structural remodelling, autonomic nervous system changes, and Ca^2+^ handling abnormalities.^40^, ^64^, ^65^, ^66^ In the current study, we mainly discussed the regulation of calcium homoeostasis by MD1 in HFpEF mice, but the relationship between MD1 and ion channels or the autonomic nervous system has not been studied. In addition, our recent study found that MD1 can regulate cardiac ion channels in pressure overload‐induced HF mice and obese mice.^17^, ^29^ Therefore, we cannot exclude the possibility that MD1 alters AF susceptibility by affecting the expression of ion channels. Second, although we used the H9C2 cell line to verify the relevant mechanism, H9C2 cells are ventricular myocytes. Given some differences between atrial myocytes and ventricular myocytes, the use of HL‐1 mouse atrial myocytes or MD1‐overexpressing mice or MD1 specific agonists may be more beneficial for elucidating the mechanism. Third, because the heart rate of normal mice is approximately 600 beats per minute, our results have shown that the heart rate was 450–500 beats per minute, which indicated that the anaesthesia maybe too stronger. Therefore, the measurement of haemodynamic and electrophysiological parameter may not be close to the values of the physiological conditions. Finally, because of the species difference between humans and mice, additional studies are needed to determine whether the regulation of vulnerability to AF by MD1 observed in a mouse model of HFpEF is likely to be beneficial in humans with HFpEF.

## Conclusions

In brief, our study demonstrated that MD1 deficiency increases the vulnerability of HFpEF mice to AF. This is mainly caused by exacerbated maladaptive LA fibrosis and inflammation of the TLR4/CaMKII signalling pathway to some extent. These findings further suggest that MD1 may be a potential therapeutic target for reducing the susceptibility of patients with HFpEF to AF.

## Conflict of interest

None declared.

## Funding

This work was supported by grants from the National Natural Science Foundation of China (81570306) and National Key R&D Program of China (2017YFC1700504).

## Supporting information


**Data S1.** Supporting Information.
