# Supplementary material for: Loss of myeloid differentiation protein 1 promotes atrial fibrillation in heart failure with preserved ejection fraction
Source: ESC Heart Fail. 2020 Jan 29;7(2):626–38. doi: 10.1002/ehf2.12620 (PMC7160510; doi:10.1002/ehf2.12620)
Supplement: Supplementary file 1 — Data S1. Supporting Information. [file EHF2-7-626-s001.docx]

**Supplementary materials**

**1 Supplementary Methods**

**1.1 The measurement of** **echocardiography**

Transthoracic echocardiography was performed at the end of 4 weeks using a Mylab30CV (ESAOTE) ultrasound system with a 15Mz probe. Cardiac measurements included an examination of LA diameter (LAD), left ventricular end-diastolic dimension (LVEDd), left ventricular end-systolic diameter (LVESd), left ventricular fractional shortening (LVFS), and left ventricular ejection fraction (LVEF).

**1.2 Invasive** **Hemodynamic Measurements (PV-Loops)**

A microtip catheter transducer (SPR-839; Millar Instruments) was inserted into the right carotid artery and advanced into the LV. After allowing the animal to stabilize for 15 min, an ARIA pressure-volume conductance system connected to the [PowerLab](http://han.medunigraz.at/han/pubmed/www.adinstruments.com/?utm_source=APS&utm_medium=etoc&utm_campaign=2010)/4SP A/D converter that stored and displayed data on a personal computer was used to continuously record pressure signals and heart rate, as previously described ^[^[^1^](#_ENREF_1)^,^ [^2^](#_ENREF_2)^]^. Parameters of systolic and diastolic function including left ventricular end-systolic pressure (LVESP), left ventricular end-diastolic pressure (LVEDP), left ventricular end-systolic volume (LVESV), left ventricular end-diastolic volume (LVEDV), stroke volume (SV), cardiac output (CO), arterial elastance (Ea) as measure of ventricular afterload, EF, Stroke work (SW), maximal slope of left ventricular systolic pressure increment (dP/d*t*_max_), isovolumetric relaxation constant Tau and maximal slope of diastolic pressure decrement (dP/d*t*_min_) were measured and calculated according to standard formulas.

**1.3 Serum indexes, organ weight, and tissue analysis**

Mice were sacrificed four weeks after saline or aldosterone infusion, and blood was obtained to determine serum aldosterone levels (LE-08604, Lai Er Bio-Tech, China). At the end of the experiment, the hearts and lungs of the sacrificed mice were dissected and weighed to compare the HW/BW (in mg/g), LW/BW (in mg/g), and HW/TL (in mg/cm) ratios. In addition, isolated LA was fixed in 4% paraformaldehyde solution, embedded in paraffin. Three-micrometer-thick sections was stained with Masson to examine collagen deposition. The ratio of the fibrotic area to the entire heart area was calculated using Image J.

**1.4 Electrocardiograph analysis**

Mice were lightly anesthetized using inhaled isoflurane (1.5-2% isoflurane). The electrodes were positioned subcutaneously to approximate surface-lead ECG (lead II). We recorded the heart rate (RR interval), P wave duration, PR, QRS, and QTc interval (in ms) among the four groups. QT interval was corrected with the modified Bazett**^’^**s formula ^[^[^3^](#_ENREF_3)^]^. Data were analyzed using Lab-Chart 7 Pro (AD Instruments).

**1.5 Electrophysiological studies**

Langendorﬀ-perfused hearts were prepared according to our previously published methods ^[^[^4^](#_ENREF_4)^]^. In brief, electrophysiological studies in isolated perfused hearts were conducted using the Langendorff apparatus with HEPES-buffered Tyrode’s solution (130 mM NaCl; 5.4 mM KCl; 1.8 mM CaCl2; 1mM MgCl2; 0.3 mM Na2HPO4; 10 mM HEPES; 10 mM glucose; pH adjusted to 7.4 with NaOH), bubbled with 95% O2–5% CO2 at 37°C and at a constant pressure of 60 mmHg to evaluate the induction of AF, interatrial conduction time (IACT), and atrial effective refractory periods (ERP). Langendorff-perfused hearts were stimulated with a pair of electrodes placed on the right atrium (RA). All isolated hearts were stabilized for 20 minutes by perfusion at a constant ﬂow before programmed electric stimulation. The hearts that did not recover to a regular spontaneous rhythm or had irreversible myocardial ischemia were discarded.

Teﬂon-coated (except at the tips) silver bipolar electrodes were placed on the appendages of the RA, LA, and left ventricle (LV). The interelectrode distance between the RA and LA was set at 5 mm to measure the IACT. The ERPs of the left and right atria were measured by the S2 extra-stimulus method using eight regularly paced beats with cycle lengths of 100, 80, 60, and 40 ms. , was measured during RA pacing. AF inducibility was tested by burst pacing methods. The induction of AF was tested by applying three chains of a 2-second burst pacing using the electronic stimulator. Specifically, the first 2-second burst had a cycle length (CL) of 40 ms (pulse duration = 5 ms). Following 3 minutes of stabilization, the second 2-second burst was applied with a CL of 20 ms (pulse duration = 5 ms). After 3 minutes of stabilization, the last 2-second burst with a CL of 20 ms was applied with a 10 ms pulse duration. AF was defined as a rapid and irregular atrial rhythm with irregular RR intervals lasting at least 1 second. The duration of AF was measured from the end of burst pacing to the first P wave detected after the rapid irregular atrial rhythm.

**1.6 Cell culture**

The rat cardiomyocyte cell line H9c2 was purchased from the Cell Bank of the Chinese Academy of Sciences and cultured in Dulbecco Modified Eagle’s Medium (DMEM) (Gibco; Thermo Fisher Scientific, Inc.) supplemented with 10% fetal bovine serum (FBS) (Gibco; Thermo Fisher Scientific, Inc.), 2 mmol/l glutamine, 1 mmol/l pyruvate, 100 U/ml penicillin and 100 mg/ml streptomycin. Cells were maintained in a humidified incubator at 37˚C and 5% CO_2_.

**1.7 Adenoviral vector infection**

Short hair‐pin RNAs (shRNAs) for MD1 knockdown and the negative control shRNA (shRNA) constructs purchased from HanBio (Shanghai, China) were used to generate MD1 knockdown adenovirus (Ad‐shMD1) and control (Ad‐shRNA) respectively. We infected H9C2 with Ad‐shMD1 and Ad‐shRNA at a multiplicity of infection of 100. And then, the cells were subsequently treated with aldosterone dissolved in dimethyl sulfoxide (DMSO) at a final concentration of 1 μM for 18 hours^[^[^5^](#_ENREF_5)^,^ [^6^](#_ENREF_6)^]^. Besides, cultured H9C2 cells that had been previously infected with Ad-shMD1 were exposed to a CaMKII inhibitor, KN93, and then treated with aldosterone for 18 hours. Protein expression levels in the treated and control cells were analyzed by western blot analysis.

**1.8 Western blotting** **analysis**

Total proteins were extracted from LA tissues using lysis buffer containing protease and phosphatase inhibitors. Extracted proteins were subjected to centrifugation, sonication and heat denaturation. Total protein concentration was determined using the BCA Protein Assay kit (cat. no. P0010; Beyotime Institute of Biotechnology). Proteins (40 μg/lane) were separated by 8%-10% SDS-PAGE and transferred onto a polyvinylidene fluoride membrane, which was subsequently incubated with primary antibodies at 4˚C. Following primary antibody incubation, the membrane was incubated with secondary antibodies for 2 h at room temperature. The antibodies used in the current study are presented in **Table S1**. Protein bands were detected using chemiluminescence (cat. no. NCI 5079; Thermo Fisher Scientific, Inc.).

**2 Supplementary Tables**

Table S1 Mouse primary antibodies for Western-blots.

| primary antibodies | Source organism | producer | Number |
| --- | --- | --- | --- |
| MD1 | Rabbit | LSBIO | LS-B6457-50 |
| Collagen I | Rabbit | abcam | ab123543 |
| Collagen III | Rabbit | absin | abs117514 |
| TGFβ1 | Mouse | abcam | ab84814 |
| TNFα | Rabbit | abcam | ab66579 |
| IL-6 | Mouse | santa | sc-57315 |
| IL-1β | Rabbit | abcam | ab82558 |
| TLR4 | Rabbit | abcam | ab13556 |
| P-p65 | Rabbit | CST | #3031 |
| p65 | Rabbit | CST | #8242 |
| P-IκBα | Rabbit | CST | #2859 |
| IκBα | Rabbit | CST | #9242 |
| p-RyR2 (Ser 2808) | Rabbit | abcam | ab59225 |
| p-RyR2 (Ser 2814) | Rabbit | badrilla | A010-31 |
| RyR2 | Mouse | abcam | ab2868 |
| SERCA2a | Mouse | abcam | ab2861 |
| p-PLB (Thr 17) | Rabbit | affbiotech | AF7278 |
| PLB | Rabbit | affbiotech | DF7726 |
| NCX1 | Mouse | abcam | ab2869 |
| p-CaMKII | Rabbit | absin | abs131059 |
| CaMKII | Rabbit | abcam | ab134041 |
| GAPDH | Rabbit | abcam | ab37168 |

**3 References**

[1] Pacher P, et al. Measurement of cardiac function using pressure-volume conductance catheter technique in mice and rats. Nature Protocols, 2008, 3(9): 1422-34

[2] Li H, et al. Regulator of G protein signaling 5 protects against cardiac hypertrophy and fibrosis during biomechanical stress of pressure overload. Proc Natl Acad Sci U S A, 2010, 107(31): 13818-23

[3] Zhang Z, et al. Functional roles of Cav1.3(alpha1D) calcium channels in atria: insights gained from gene-targeted null mutant mice. Circulation, 2005, 112(13): 1936-44

[4] Shuai W, et al. MD1 Deficiency Promotes Inflammatory Atrial Remodelling Induced by High-Fat Diets. Canadian Journal of Cardiology, 2019, 35(2): 208-16

[5] Garcia AG, et al. Interferon-gamma ablation exacerbates myocardial hypertrophy in diastolic heart failure. Am J Physiol Heart Circ Physiol, 2012, 303(5): H587-96

[6] Tanaka K, et al. Follistatin like 1 Regulates Hypertrophy in Heart Failure with Preserved Ejection Fraction. JACC Basic Transl Sci, 2016, 1(4): 207-21
